# Maternal mental health and infant neurodevelopment at 6 months in a low‐income South African cohort

**DOI:** 10.1002/imhj.22021

**Published:** 2022-10-21

**Authors:** Marlette Burger, Christa Einspieler, Dana J. H. Niehaus, Marianne Unger, Esme R. Jordaan

**Affiliations:** ^1^ Faculty of Medicine and Health Sciences Physiotherapy Division, Department of Health and Rehabilitation Sciences Stellenbosch University Stellenbosch South Africa; ^2^ Research Unit iDN––Interdisciplinary Developmental Neuroscience, Division of Phoniatrics Medical University of Graz Graz Austria; ^3^ Faculty of Medicine and Health Sciences Department of Psychiatry, Stellenbosch University Stellenbosch South Africa; ^4^ Biostatistics Unit South African Medical Research Council Parow South Africa; ^5^ Statistics and Population Studies University of the Western Cape Cape Town South Africa

**Keywords:** infant, maternal mental health disorders, maternal psychosis, neurodevelopment, persistent mental health disorders, South Africa, trastornos de salud mental materna, trastornos persistentes de salud mental materna, sicosis materna, neurodesarrollo del infante, Sudáfrica, troubles de la santé mentale maternelle, troubles persistants de la santé mentale maternelle, psychose maternelle, neurodéveloppement du nourrisson, Afrique du Sud, Psychische Störungen bei Müttern, Anhaltende psychische Störungen bei Müttern, Psychose bei Müttern, neurologische Entwicklung von Säuglingen, Süd‐Afrik, 「母親の精神疾患」「母親の持続的な精神疾患」「母親の精神病」「乳幼児の神経発達」「南アフリカ共和国」, 孕产妇心理健康障碍, 持续性孕产妇心理健康障碍, 孕产妇精神病, 婴儿神经发育, 南非, **الكلمات المفتاحية**: “اضطرابات صحة الأم النفسية” ، “اضطرابات الصحة النفسية للأم المستمرة” ، “ذهان الأم” ، “النمو العصبي للرضع” ، “جنوب إفريقيا

## Abstract

Maternal mental health disorders and the adverse consequences for infant neurodevelopment have received substantial research attention in high‐income countries over the past five decades. In Africa, where relatively little work has been done on this topic, researchers have largely focused on infant physical health outcomes. This longitudinal study investigated the neurodevelopment of infants at 6 months post‐term with exposure to mothers with a clinical diagnosis of persistent mental health disorders residing in low‐income communities in Cape Town, South Africa. Adjusted models revealed no significant differences on the Bayley Scales of Infant and Toddler Development (BSID‐III) domains (cognitive, motor, language, socio‐emotional, and adaptive behavior) between infants exposed to maternal mental health disorders (*n* = 62) and the comparison group (*n* = 35) at 3 and 6 months. Subgroup analyses found no significant differences on the BSID‐III domains between infants with exposure to mood disorders (*n* = 31), as well as infants with exposure to comorbid (i.e., a combination of two or three) mental health disorders (*n* = 14) and the comparison group. However, infants with exposure to psychotic disorders (*n* = 14) scored significantly lower on the cognitive and the motor domains and the fine motor subscale. These novel data provide an important contribution to the scientific literature especially in the field of maternal psychotic disorders in Africa.

## Resumen

Los trastornos de la salud mental materna y las consecuencias adversas para el neurodesarrollo del infante han recibido una considerable atención investigativa en países de altos niveles económicos a lo largo de las últimas cinco décadas. En África, donde se ha llevado a cabo relativamente poco trabajo sobre este tema, los investigadores se han enfocado por la mayor parte en los resultados de la salud física del infante. Este estudio longitudinal investigó el neurodesarrollo de infantes a los 6 meses después del término de gestación que habían sido expuestos a madres con un diagnóstico clínico de trastornos de salud mental persistentes quienes residían en comunidades de bajos recursos en Ciudad del Cabo, Sudáfrica. Los ajustados modelos no revelaron significativas diferencias en los dominios de las Escalas Bayley del Desarrollo del Infante y Niños Pequeñitos (BSID‐III) (cognitivo, motor, lenguaje, comportamiento socioemocional y de adaptación) entre los infantes que habían estado expuestos a los trastornos de salud mental materna (n = 62) y el grupo de comparación (n = 35) a los 3 y 6 meses. Los análisis de subgrupo no encontraron diferencias significativas en los dominios de BSID‐III entre los infantes que habían estado expuestos a los trastornos de estado de ánimo (n = 31), así como los infantes que habían estado expuestos a trastornos de salud mental comórbidos (v.g. una combinación de dos o tres) (n = 14) y el grupo de comparación. Sin embargo, los infantes que habían estado expuestos a trastornos sicóticos (n = 14) tuvieron puntajes significativamente más bajos en los dominios cognitivo y motor, así como en la subescala de las habilidades motoras finas. Estos novedosos datos ofrecen una contribución importante a la literatura científica especialmente en el campo de los trastornos sicóticos maternos en África.

## Résumé

Les troubles de la santé mentale maternelle et les conséquences négatives pour le neurodéveloppement du nourrisson ont reçu l'attention de beaucoup de recherches dans les pays à revenu élevé ces cinquante dernières années. En Afrique où relativement peu de travail a été fait sur ce sujet, les chercheurs se sont en grande partie penchés sur les résultats de la santé physique des nourrissons. Cette étude longitudinale s'est penchée sur le neurodéveloppement de nourrissons à 6 mois après terme avec une exposition aux mères avec un diagnostic clinique de troubles de la santé mentale persistants, résidant dans des communautés défavorisées à Cape Town, en Afrique du Sud. Les modèles ajustés n'ont révélé aucunes différences importantes dans les domaines (cognitif, moteur, langage, comportement socio‐émotionnel et comportement adaptif) des Echelles Bayley du Développement du Nourrisson et du Jeune Enfant (BSID‐III) entre les nourrissons exposés à des troubles de la santé mentale maternelle (n = 62) et le groupe de comparaison (n‐35) à 3 et 6 mois. Les analyses de sous‐groupes n'ont trouvé aucunes différences concernant les domaines BSID‐III entre les nourrissons avec une exposition à des troubles de l'humeur (n‐31), ainsi que des nourrissons avec une exposition à des troubles de santé mentale comorbides (c'est‐à‐dire une combinaison de deux ou trois) (n‐14) et le groupe de comparaison. Cependant, les nourrissons ayant été exposés à des troubles psychotiques (n = 14) ont reçu des scores bien plus bas dans le domaine cognitif, dans le domaine moteur, et à la sous‐échelle motrice fine. Ces nouvelles données offrent une contribution importante aux recherches scientifiques, surtout dans le domaine des troubles psychotiques maternels en Afrique.

## Zusammenfassung

Psychische Gesundheit von Müttern und neurologische Entwicklung von Säuglingen im Alter von 6 Monaten in einer südafrikanischen Kohorte mit niedrigem Einkommen

Psychische Störungen bei Müttern und die negativen Folgen für die neurologische Entwicklung ihrer Säuglinge haben in der Forschung in den letzten fünf Jahrzehnten in Ländern mit hohem Einkommen große Beachtung gefunden. In Afrika, wo relativ wenig zu diesem Thema geforscht wurde, haben sich Forschende bislang weitgehend auf die körperliche Gesundheit der Kinder konzentriert. In dieser Längsschnittstudie wurde die neurologische Entwicklung von Säuglingen aus einkommensschwachen Gemeinden in Kapstadt, Südafrika, untersucht, deren Mütter mit mind. einer anhaltenden psychischen Störung diagnostiziert wurden. Der Erhebungszeitraum erstrechte sich bis 6 Monate nach der Geburt. Modelle mit angepassten Werten zeigten 3 und 6 Monate nach der Geburt keine signifikanten Unterschiede in den Domänen der „Bayley Scales of Infant and Toddler Development“ (BSID‐III; kognitives, motorisches, sprachliches, sozio‐emotionales und adaptives Verhalten) zwischen Säuglingen von Müttern mit psychischen Störungen (n = 62) und der Vergleichsgruppe (n = 35). Subgruppenanalysen ergaben keine signifikanten Unterschiede in den BSID‐III‐Domänen zwischen Säuglingen von Müttern mit einer affektiven Störung (n = 31) sowie Säuglingen von Müttern mit komorbiden (d.h. einer Kombination von zwei oder drei) psychischen Störungen (n = 14) und der Vergleichsgruppe. Allerdings schnitten Säuglinge von Müttern mit psychotischen Störungen (n = 14) in der kognitiven und motorischen Domäne sowie in der feinmotorischen Subskala signifikant schlechter ab. Diese neuen Daten stellen einen wichtigen Beitrag zur wissenschaftlichen Literatur dar, insbesondere auf dem Gebiet der psychotischen Störungen bei Müttern in Afrika.

## 抄録

南アフリカの低所得者コホートにおける月齢6ヵ月時の母親のメンタルヘルスと乳児の神経発達

母親の精神疾患と乳児の神経発達への悪影響は、過去50年間、高所得国においてかなりの研究の関心を集めてきた。このテーマに関する研究が比較的少ないアフリカでは、研究者は主に乳幼児の身体的健康の結果に焦点をあててきた。この縦断研究では、南アフリカ共和国のケープタウンの低所得者地域に住む、持続性な精神疾患と臨床診断を受けた母親に晒された、生後6ヶ月の乳児の神経発達を調査した。調整モデルにより、母親の精神疾患に晒された乳児 (n = 62) と比較群 (n = 35) の間で、3ヶ月時と6ヶ月時のベイリー乳幼児発達尺度 (BSID‐III) の領域 (認知、運動、言語、社会情動、適応行動) に有意差はなかった。サブグループ解析の結果、気分障害に晒された乳児 (n = 31) 、および併存する (2つまたは3つの) 精神疾患に晒された乳児 (n = 14) と比較群の間で、BSID‐IIIドメインに有意差は認められなかった。しかし、精神病性障害に晒された乳児 (n = 14) は、認知領域と運動領域、微細運動下位尺度で有意に低い値を示した。これらの新しいデータは、特にアフリカの母親の精神病性障害の分野において、科学文献に重要な貢献をするものである。

## 摘要

过去50年来, 高收入国家对孕产妇心理健康障碍及其对婴儿神经发育的不利影响进行了大量研究。在非洲, 这方面的研究相对较少, 研究人员主要关注婴儿的身体健康状况。这项纵向研究调查了居住在南非开普敦低收入社区且临床诊断为持续性心理健康障碍的母亲所接触的婴儿在产后6个月的神经发育情况。调整后的模型显示, 在3个月和6个月时, 暴露于母亲心理健康障碍的婴儿 (n = 62) 和对照组 (n = 35) 之间, 在“贝利婴幼儿发展量表” (BSID‐III) 领域 (认知、运动、语言、社交情感和适应行为) 没有显著差异。亚组分析发现, 暴露于情绪障碍的婴儿 (n = 31) 以及暴露于共病 (即两种或三种的组合) 心理健康障碍的婴儿 (n = 14) 和对照组之间, 在BSID‐III领域没有显著差异。然而, 暴露于精神病性障碍的婴儿 (n = 14) 在认知和运动领域以及精细运动分量表上的得分明显较低。这些新数据为科学文献, 特别是为非洲孕产妇精神病性障碍领域, 提供了重要的贡献。

## ملخص

صحة الأم النفسية والنمو العصبي للرضع في 6 أشهر في مجموعة جنوب أفريقيا منخفضة الدخل

حظيت اضطرابات الصحة النفسية للأمهات والعواقب السلبية على النمو العصبي للرضع باهتمام بحثي كبير في البلدان ذات الدخل المرتفع على مدى العقود الخمسة الماضية. في إفريقيا حيث تم القيام بعمل قليل نسبيًا حول هذا الموضوع ، ركز الباحثون إلى حد كبير على نتائج الصحة البدنية للرضع. استقصت هذه الدراسة الطولية النمو العصبي للرضع في عمر 6 أشهر مع التعرض للأمهات اللائي لديهن تشخيص إكلينيكي لاضطرابات الصحة النفسية المزمنة والمقيمات في مجتمعات منخفضة الدخل في كيب تاون ، جنوب أفريقيا. كشفت النماذج المعدلة عن عدم وجود فروق ذات دلالة إحصائية في مقاييس بايلي لمجالات نمو الرضع والأطفالIII) ‐(BSID (السلوك المعرفي والحركي واللغوي والاجتماعي والعاطفي والتكيفي) بين الرضع المعرضين لاضطرابات صحة الأم النفسية (ن = 62) و مجموعة المقارنة (ن = 35) عند 3 و 6 أشهر. لم تجد تحليلات المجموعات الفرعية فروقًا ذات دلالة إحصائية في مجالاتIII ‐BSID بين الرضع الذين يعانون من اضطرابات المزاج (ن = 31) ، وكذلك الرضع الذين تعرضوا لاضطرابات مرضية مشتركة (أي مزيج من اثنين أو ثلاثة) من اضطرابات الصحة النفسية (العدد = 14) ومجموعة المقارنة. ومع ذلك ، فإن الأطفال الذين تعرضوا لاضطرابات ذهانية (ن = 14) سجلوا درجات أقل بشكل ملحوظ في المجال المعرفي والحركي والمقياس الفرعي للحركة الدقيقة. توفر هذه البيانات الجديدة مساهمة مهمة في الأدبيات العلمية خاصة في مجال الاضطرابات الذهانية لدى الأمهات في إفريقيا.

## 1 INTRODUCTION

Healthy child development is essential for a thriving and productive society with a sustainable future. Child health and development are at the heart of the 2030 Global Agenda, and there is growing recognition by policymakers that improving child survival rates in low‐ and middle‐income countries (LMICs) is no longer sufficient (Bhardwaj et al., 2017). Children should not merely survive but also thrive in order to develop their full potential (Bhardwaj et al., 2017; Black et al., 2017). To thrive, children need nurturing care and a stable and supportive environment (Black et al., 2017). Optimal child development is multi‐faceted and requires many role‐players to work together. In LMICs, the primary caregiver, mainly the mother, is the fundamental role‐player in infant survival, wellbeing, and development (Parsons et al., 2012). During the first postnatal year, day‐to‐day maternal–infant interactions provide early learning opportunities and influence cognitive, language, emotional and social‐behavioral development throughout childhood (Black et al., 2017; Parsons et al., 2012). Since the environment is more oppressive in LMICs than in high‐income countries (HICs), the quality of caregiving during this critical time frame might play a greater role in an infant's physical wellbeing and neurodevelopment (Stein et al., 2014). Burdens such as single parenthood, unemployment, low education, poverty, lack of adequate nutrition, overcrowding, lack of electricity, clean running water, and poor sanitation are common. Furthermore, mothers are also exposed to gender‐based violence, alcohol and drugs, and infectious diseases such as HIV/AIDS and tuberculosis (Black et al., 2017; Stein et al., 2014; Parsons et al., 2012). In these adverse environments, new mothers have to practice self‐care, be sensitive and responsive to the infant's emotional and physical needs, initiate and maintain exclusive breastfeeding, maintain good hygiene, obtain appropriate health care for the infant, for example, regular well‐baby clinic visits and immunizations for infants (Parsons et al., 2012).

**KEY FINDINGS**

1. Maternal mental subgroup disorder analyses revealed no significant differences in the BSID‐III domains and subscales between infants with exposure to persistent maternal mood disorders and infants with exposure to persistent maternal comorbid mood, anxiety, and psychotic disorders at 3 and 6 months compared to infants with no exposure.
2. Infants with exposure to persistent maternal psychotic disorders at 3 and 6 months scored significantly lower on the cognitive and motor domains and the fine motor subscale of the BSID‐III than infants with no exposure to maternal mental health disorders.
3. This study has shown that despite being exposed to substantial maternal mental health adversities and living in poor socio‐economic status settings, the majority of infants in our study did not score significantly lower on the different domains and subscales of the BSID‐III at 6 months compared to infants with no exposure.

**RELEVANCE**

The current study is among the first to report on the early neurodevelopmental outcome among infants exposed to a clinical diagnosis of persistent maternal mental health disorders. Only infants with exposure to psychotic disorders at 3 and 6 months scored significantly lower on the cognitive and motor domains of the BSID‐III. This novel data provides an important contribution to the scientific literature given the lack of published data, especially in the field of maternal psychotic disorders in Africa.

The accumulation of these adversities faced in LMICs may have a detrimental impact on early human development and could impede the infant's psychosocial, cognitive, and physical development (Trude et al., 2021). A series of Lancet articles on early childhood development, published in 2017, found that approximately 250 million children younger than five years and living in LMICs were at risk of not reaching their developmental potential (Black et al., 2017). However, recent evidence from Brazil and South Africa suggests that nurturing care may modify the negative effects of cumulative adversities on early childhood development (Trude et al., 2021). Undeniably, the mother's physical and psychological wellbeing plays an essential role in the quality of nurturing care during the first postnatal year (Parsons et al., 2012). Maternal mental health disorders not only affect women's overall wellbeing but has serious inter‐generational consequences. Poor maternal mental health can reduce effective and responsive maternal caregiving behaviors (Miklush & Connelly, 2013), which compromised the mother–infant relationship and may profoundly affect the infant's environment and neurodevelopment (Shonkoff et al., 2012).

The World Health Organization (WHO) indicated that maternal mental health disorders in LMICs are a serious but under‐recognized public health concern (World Health Organization, 2008). Maternal mental health disorders and the adverse consequences for mother–infant relationships and child health and development have received substantial research attention in HICs over the past five decades (Burger et al., 2020; Fisher et al., 2011). It is only since the start of the 21st century that there has been an increase in published research on maternal mental health disorders in Africa (Ng'oma et al., 2020). However, the main focus has been on perinatal complications and infant physical health outcomes, such as birth weight and physical growth (Parsons et al., 2012). A recent position statement by the World Psychiatric Association appeals for research on perinatal mental disorders to be conducted across the diagnostic spectrum, including the impact on early childhood development (World Psychiatric Association, 2017). Mothers experiencing persistent mental health disorders (as measured at more than one time point) are expected to encounter considerably more difficulties with parenting and maternal–infant interactions than mothers with brief episodes of mental health disorders (Sohr‐Preston & Scaramella, 2006). In fact, a key factor believed to moderate the effect of postnatal maternal mental health disorders on child outcomes is the severity and particularly the persistence of mental health disorders during the postnatal period. A systematic review by Stein et al. (2014) found that low socio‐economic status, the absence of social and partner support, and the persistence of maternal mental health disorders increase the risk of adverse infant developmental outcomes. However, in the absence of social adversities and severe or persistent maternal mental health disorders (e.g., short duration of maternal mental health disorders), the risks to the infant are generally low (Stein et al., 2014).

The primary aim of this paper is to describe the neurodevelopment of infants at 6 months post‐term with exposure to mothers with a clinical diagnosis of persistent mental health disorders residing in low‐income communities in Cape Town, South Africa. The objectives of the study are (i) to describe the sociodemographic and maternal–infant health characteristics, (ii) the maternal mental health diagnoses at 3 and 6 months, and (iii) to explore associations between persistent maternal mental health disorders at 3 and 6 months and infant cognitive, motor, language, social‐emotional and adaptive behavior at 6 months corrected age after adjusting for sociodemographic variables and maternal–infant health outcomes. Subgroup analyses will also be conducted to determine the association of mothers with mood disorders at 3 and 6 months; psychotic disorders at 3 and 6 months; and comorbid (i.e., a combination of two or three) mental health disorders at 3 and/or 6 months on infant neurodevelopment at 6 months post‐term.

## 2 METHODS

### 2.1 Study design and setting

The present study is a nested longitudinal study within a larger ongoing longitudinal cohort design, the Maternal and Infant Mental Health (MIMH) study. The MIMH study is based at Stikland Psychiatric Hospital, a tertiary state‐owned specialist psychiatric facility providing psychiatric in and outpatient services to predominantly lower socio‐economic urban and peri‐urban communities in the northern suburbs of Cape Town. In the MIMH study, females, 18 years and older, with a previous or current psychiatric diagnosis, attending Stikland Maternal Mental Health Outpatient Clinic were enrolled during the 2nd or 3rd trimester of pregnancy and are followed up prospectively until their children are 4 years old. Women attending Stikland Maternal Mental Health Outpatient Clinic were predominantly of mixed ancestry (Afrikaans or English speaking) or Black Africans (isiXhosa or English speaking).

### 2.2 Procedure

As part of the MIMH study, trained study staff collected an extensive record of maternal and infant peri‐ and postnatal health data from hospital records, the infants’ Road to Health booklets (Western Cape Government, 2019) and interviewing the mothers. Sociodemographic variables were collected antenatally and reviewed at the 6‐month postnatal visits. At the 6‐months’ assessment, infants' weight and maternal reports and hospital records of infant health since birth were collected. These included reasons for hospitalization since birth, that is, lower respiratory tract infections and bacterial meningitis.

### 2.3 Description of mother–infant participants

Infants born to mothers with a history of psychiatric disorders, attending Stikland Maternal Mental Health Outpatient Clinic between April 1st, 2014 and September 30th, 2019, were eligible for inclusion in the current study. We included infants exposed to mothers with a diagnosis of psychiatric illness according to the Diagnostic and Statistical Manual of Mental Disorders‐V (DSM‐V) (Regier et al., 2013) at two time points, namely 3‐ and 6‐month post‐term. Infants with prenatal or postnatal (through breastfeeding) exposure to psychotropic medication and medication for other medical conditions, namely epilepsy, diabetes mellitus, or human immunodeficiency virus (HIV) infection, were not excluded from the study. Infants with a higher risk for developmental delays such as low birth weight and/or born preterm, HIV exposed but not infected or HIV infected were also not excluded from the study. The rationale for not excluding these infants with a risk for developmental delays was that maternal mental health disorders are linked to unhealthy maternal lifestyles such as increased rates of smoking, alcohol and illicit drug use, inadequate selfcare, and poor attendance at antenatal clinics (Judd et al., 2014; Hartley et al., 2011). Furthermore, comprised maternal mental health is significantly associated with adverse pregnancy, birth, and infant health outcomes, including intra‐uterine growth retardation (IUGR), low birth weight, and preterm delivery in Africa (Dadi et al., 2022). South‐African women of reproductive age (aged 15–49 years) experience a high prevalence of HIV (26.3%) (Simbayi et al., 2019) and women with HIV exhibit a significantly increase in depressive symptoms during the perinatal period (Zhu et al., 2019). However, infants with the following conditions or disorders (and therefore a higher risk for developmental delays) were excluded from the study: hypoxic‐ischemic encephalopathy, birth malformations of the central nervous system (e.g., myelomeningocele), congenital disorders (e.g., arthrogryposis multiplex congenital), microcephaly (<3rd percentile for gestational age and sex), and infants diagnosed with syndromes or genetic/chromosomal defects (e.g., Down syndrome). Infants with a significant hearing or vision impairment, as screened at 14 weeks and 6 months post‐term age by health care workers at well baby clinics, were not included in the study. Infants who were placed in foster care were not eligible for the study. A comparison group of infants with no antenatal or postnatal exposure to psychiatric illnesses and psychotropic medication (or any other antenatal or postnatal pharmacological treatment or substance abuse) residing from the same low‐income geographical area were recruited by obstetric nurse practitioners at day hospitals and community health clinics. The mother–infant pairs that form part of the comparison group were only recruited after the birth of the infants, and since no records on previous maternal medical history were available, the prenatal medical and psychiatric history were based on maternal self‐report. A total of 112 mother–infant dyads were eligible for the study; three mother–infant dyads were excluded from the analysis because the mother did not receive a 3 month (*n* = 1) or a 6 month (*n* = 2) psychiatric assessment. Another 13 mother–infant pairs were excluded because the mother had a diagnosis of psychiatric disorder at one time point only, namely at 3 months post‐term (*n* = 8) or at 6 months post‐term (*n* = 5). The final study cohort consisted of 96 mother–infant dyads and included 35 infants with no exposure to maternal mental health disorders (comparison group) and 61 infants with exposure to maternal mental health disorders at 3‐ and 6‐month post‐term. Maternal mental health disorders included the following main categories of psychiatric disorders across the diagnostic spectrum: mood, anxiety, and psychotic disorders.

### 2.4 Assessment of infant neurodevelopment: Bayley Scales of Infant and Toddler Development

At 6 months post‐term age, neurodevelopment was assessed with the Bayley Scales of Infant and Toddler Development^®^, Third Edition (BSID‐III) (Bayley, 2006). The BSID‐III is a standardized, norm‐referenced tool and is seen as the gold standard amongst investigators for neurodevelopmental assessment from 0 to 42 months (Schonhaut et al., 2013). The World Bank has recommended the BSID‐III as the primary tool for the assessment of infant and toddler development in LMICs (Fernald et al., 2009). No published norms for the BSID‐III in African countries are currently available. However, the BSID‐III has been validated in healthy Black urban infants aged 2–13 months in South Africa with similar reported values to those of the BSID‐III United States‐based reference population (Rademeyer et al., 2013).

The cognitive, fine and gross motor, and expressive and receptive language scales of the BSID‐III were administered in the home language of the infants by a trained paediatric physiotherapist blinded to the maternal diagnoses and infant risk factors. The socio‐emotional and adaptive behavior domains were assessed by direct observation of the infant as well as by interviewing the caregiver. Conversion tables from the BSID‐III manual were used to convert raw developmental scores into age‐standardized composite scores for the cognitive, motor, language, socio‐emotional and adaptive behavior developmental domains (mean ± SD of 100 ± 15), while scaled scores were obtained for fine and gross motor, and expressive and receptive language subscales. According to the standard guidelines, composite scores were classified into four groups, namely, above‐average performance (standardized score > 115), average performance (standardized score 85–115), delayed performance (standardized score 70–85), and severely impaired (<70) (Bayley, 2006). Scaled scores were classified as above average (>13), average (8−12), and below‐average (≤7). Infants scoring ≤1 SD below the mean of the composite (<85) and/or scaled scores (≤7) were classified as demonstrating a clinically significant developmental delay in that domain or subscale. Unless indicated otherwise, motor development refers to combined gross and fine motor development and language development to combined expressive and receptive language development. Since infant‐rearing variations between ethnic groups may influence infant neurodevelopment, we differentiated between two major ethnic groups, namely Mixed‐Ancestry and Black African, during our analysis.

### 2.5 Assessment of maternal mental and physical health

At the 3‐ and 6‐month postnatal visits, mothers completed semi‐structured interviews and thorough, structured psychiatric assessments as per care‐as‐usual. During the semi‐structured interviews, current physical health and general medical history were assessed. These assessments were conducted by a qualified senior psychiatrist with a minimum of 5 years’ experience. Trained translators assisted in cases where participants were unable to communicate in either English or Afrikaans (e.g., women speaking Xhosa). A psychiatric diagnosis was made according to the diagnostic criteria set out in the DSM‐V (Regier et al., 2013). Mothers were grouped according to their psychiatric diagnosis as having either mood, psychotic, or anxiety disorder(s), or a combination of two or three disorders. The following tools were included in the psychiatric assessments: the Mini International Neuropsychiatric Interview (MINI) (Sheehan et al., 1998), the Edinburgh Postnatal Depression Scale (EPDS) (Cox et al., 1987), and the Recent Life Events Questionnaire (RLEQ) (Brugha et al., 1985). During the interview, substance use during pregnancy and the postnatal period was assessed with a dichotomous measure (exposure versus no exposure) to document exposure to the following: smoking (tobacco), alcohol, and recreational drugs. The frequency of substance use was not recorded.

### 2.6 Ethical considerations

The study was approved by the Health Research Ethics Committee at Stellenbosch University (S12/04/111). Permission was obtained from Stikland Hospital and the Western Cape Provincial Department of Health. Trained research assistants obtained written informed consent from mothers or legal guardians of included infants in their preferred language, namely English, Afrikaans or isiXhosa.

### 2.7 Statistical analysis

The data were entered into an Excel spreadsheet (Microsoft 2010) and then analyzed using the SAS 9.4 statistical program (SAS Institute Inc, Cary, North Carolina). Descriptive statistics for maternal demographic, psychosocial characteristics, and infant birth and 6‐month outcomes included frequencies (%) for categorical variables and mean, median, and standard deviation for numeric variables, overall and by maternal mental health status (maternal mental health disorder group, and comparison group with no history of mental health disorders). Comparisons between groups were provided using Chi‐Square tests and *t*‐tests. Frequencies of the combinations of mental health disorder diagnoses (mood, psychotic, anxiety) are reported. Infants with exposure to mothers with any of the mental health disorders at 3‐ and 6‐month post‐term were compared to infants with no exposure (comparison group). Further subgroup analyses were conducted, comparing the comparison group to groups with (i) mood disorders at 3‐ and 6 months post‐term; (ii) psychotic disorders at 3‐ and 6 months post‐term; and (iii) comorbid mood, anxiety, and psychotic disorders at 3‐ and 6 months post‐term.

The outcomes were the responses to the nine BSID‐III subtest composite and scaled scores (cognitive composite score, motor composite score, fine motor scaled score, gross motor scaled score, language composite score, receptive scaled score, expressive scaled score, social‐emotional composite score, and adaptive behavior composite score). Linear regression models (analysis of variance) were conducted for each separate outcome variable to assess the association with the mental health disorder groups and to compare the mean BSID‐III subtest score of the comparison group with the overall mental health group and each of the three defined mental health disorder subgroups. All relevant maternal–infant socio‐demographic and health covariates were considered for inclusion as confounders in the models depending on the size of their correlation with the different BSID‐III domains. The following covariates, with a correlation above .2 for most of the BSID‐III outcomes, were eligible for inclusion in the models: gestational age, average family income, ethnicity, HIV status, feeding practices, and preterm birth. However, due to small sample sizes, only gestational age was selected for inclusion in all models and was also considered as a proxy for other confounders. To report the associations, Chi‐square *p*‐values and the mean differences and 95% confidence limits were provided.

## 3 RESULTS

### 3.1 Maternal–infant sociodemographic and health characteristics

Table 1 presents the characteristics of the mothers and infants who participated. The study group consisted of 97 mother–infant dyads and included 35 infants with no exposure to maternal mental health disorders (comparison group). There were significant differences between the groups for ethnicity (*p* = .001), antenatal substance use (*p* = .001), gestational age (*p* = .023), birth weight (*p* = .001), head circumference‐for‐age *z* score (HCAZ) (*p* = .004) and feeding practices at 6 months (*p* = .046).

**TABLE 1:** Maternal‐infant sociodemographic and health characteristics

**Maternal demographic and psychosocial characteristics**	***n***	**Total number of mother‐infant dyads**	**Maternal mental health disorders**	**Comparison group**	***p*‐value**
**Number of mothers**	97	97 (100%)	*n* = 62	*n* = 35	
**Age [median/mean/SD (min–max)]**		30/30.7/5.6 (17–45)	31/31.4/5.7 (22–45)	30/29.6/5.4 (17–38)	.144
**Ethnicity**					
Black African	97	44 (45.4%)	15 (24.2%)	29 (82.9%)	.001*
Mixed ancestry		53 (54.6%)	47 (75.8%)	6 (17.1%)	
**Educational achievement**					
Secondary education^a^	97	88 (90.7%)	56 (90.3%)	32 (91.4%)	.856
Tertiary education^a^		9 (9.3%)	6 (9.4%)	3 (8.6%)	
**Antenatal substance use**					
None	96	68 (70.8%)	39 (62.9%)	29 (85.3%)	.001*
Tobacco smoking		15 (15.6%)	15 (24.2%)	0	
Alcohol/Recreational drugs		13 (13.6%)	8 (12.9%)	5 (14.7%)	
**Antenatal physical illness**					
HIV+	97	14 (14.4%)	7 (11.3%)	7 (20%)	.249
HIV−		83 (85.6%)	55 (88.7%)	28 (80%)	
**Current substance use (6 months)**					
None	94	56 (59.6%)	31 (51.7%)	25 (73.5%)	.045
Tobacco smoking		20 (21.3%)	17 (28.3%)	3 (8.8%)	
Alcohol/Recreational drugs		18 (19.2%)	12 (20.0%)	6 (17.7%)	
**Marital status (6 months)**					
Married/Cohabiting	94	41 (43.6%)	26 (43.4%)	15 (44.1%)	.941
Separated/Divorced/Single		53 (56.4%)	34 (56.6%)	19 (55.9%)	
**Employed (6 months)**					
Employed	91	27 (29.7%)	17 (28.8%)	10 (31.2%)	.809
Unemployed		64 (70.3%)	42 (71.2%)	22 (68.8%)	
**Average family income (per month at 6 months)**					
<300 US$	93	87 (93.5%)	55 (93.2%)	32 (94.1%)	.865
>300 US$		6 (6.5%)	4 (4.3%)	2 (5.9%)	
**Infant birth outcomes**					
**Gender: Male**	97	53 (54.6%)	34 (54.8%)	19 (54.3%)	.958
**Gender: Female**		43 (44.8%)	28 (45.2%)	16 (45.7%)	
**Gestational age**: median/mean/SD (min–max)	97	39/38.4/2.2 (27–42)	39/38.4/2.2 (27–42)	39/39.3/1.3 (37–42)	.023*
**Preterm birth (<37 weeks)**	97	7 (7.2%)	7 (11.3%)	0	
**Birth weight, g**: median/mean/SD (min–max)	97	3090/3130/519.5 (1285–4805)	2985/3000/482 (1285–4000)	3185/3361/510.3 (2590–4805)	.001*
**APGAR score at 5 min** median/mean/SD (min–max)	95	10/9.5/.9 (4–10)	10/9.4/1.0 (4–10)	10/9.6/.7 (7–10)	.558
**Infant outcomes at 6 months post‐term**					
Weight: Female (kg) median/mean/SD (min–max)	32	8.0/7.8/.87 (6.2–9.6)	7.7/7.6/.85 (6.4–9.0)	8.0/8.2/.9 (6.2–9.6)	.982
Weight: Male (kg) median/mean/SD (min–max)	44	8.0/8.2/.86 (6.6–11.2)	8.0/8.2/.98 (6.6–11.2)	8.0/8.2/.7 (7–9.2)	.082
WAZ median/mean/SD (min–max)	76	.4/.3/.99 (−1.9–3.2)	.19/.22/1.0 (−1.9–3.2)	.6/.5/.9 (−1.4–2.2)	.253
Low WAZ (WAZ of −2 or below)	76	0	0	0	
Head circumference (cm) median/mean/SD (min–max)	97	44/44.2/1.5 (39–49)	44/43.9/1.5 (39–46.5)	45/44.8/1.5 (42–49)	
HCAZ median/mean/SD (min–max)	97	1.0/1.1/1.2 (−2.99–4.98)	.79/.83/1.1 (−2.99–2.97)	1.7/1.5/1.1 (−.5–5.0)	.004*
Low HCAZ (HCAZ of −2 or below)	97	1	1	0	
**Current feeding practices (6 months)**					
Breastfeeding and solids	94	28 (29.8%)	14 (23.3%)	14 (41.2%)	.046*
Formula Feeding and solids		49 (52.1%)	37 (61.7%)	12 (35.3%)	
Mixed feeding and solids		17 (18.1%)	9 (15.0%)	8 (23.5%)	
**Current caregiver during the day (6 months)**					
Mother only	94	82 (87.2%)	52 (86.7%)	30 (88.2%)	.826
Mother and other (grandmother; nanny; crèche)		12 (12.8%)	8 (13.3%)	4 (11.8%)	

Abbreviations: HCAZ, head circumference‐for‐age z score; HIV, human immunodeficiency virus; kg, kilogram; SD, standard deviation; US$, United States dollars; WAZ, weight‐for‐age z score.

^a^Completed secondary or tertiary education.

^*^ *p*‐values: <.05.

Of the total group, 14.4% of the mothers were diagnosed with HIV during their pregnancy; however, none of their infants were HIV‐infected. Seven infants (7.2%) were born preterm. One infant (with exposure to a mood disorder) was born extremely preterm (gestational age: 27 weeks; birthweight 1285 g), while six infants were born moderate‐to‐late preterm (gestational age range: 34–36 weeks). None of the infants in the comparison group were born before 37 weeks gestation. Three infants (2.8%) exposed to maternal mental health disorders were small for gestational age. Four infants exposed to maternal mental health disorders had a history of postnatal hospitalization during the first 3 post‐term months. One infant was diagnosed with a lower tract respiratory infection, one with an Escherichia coli infection, and two with jaundice. One infant in the comparison group was diagnosed with acute bacterial meningitis at 8 weeks post‐term. None of the infants were hospitalized between 3 and 6 months post‐term. At 6 months, only one infant (with exposure to maternal mental health disorders) had a HCAZ below −2, while none of the infants had a low weight‐for‐age *z* score (WAZ). At the 6‐month postnatal assessment, 87.2% of the mothers were looking after their infants during the day (e.g., infants did not attend a day‐care facility). Of the 29.7% of mothers who were employed, 16.9% were employed in the informal sector or self‐employed (e.g., street vendors, domestic workers, selling goods from their home, or waste pickers) and were, therefore, able to look after their infants during the day while they were working. A total of 12 mothers (12.8%) had to return to the formal employment sector after the end of their 3‐month maternity leave. Their infants attended a crèche or the grandmother or a nanny were looking after the infants during the day (Table 1).

### 3.2 Maternal mental health diagnosis at 3 and 6 months postnatal age

Table 2 presents the psychiatric diagnoses of the mothers at 3 and 6 months postnatal age. Mothers were grouped according to their psychiatric diagnosis as having either a mood, psychotic, or anxiety disorder, or a combination of two or three disorders. The most common diagnosis at both time points was mood disorders (*n* = 31), while 14 mothers were diagnosed with psychotic disorders. Only three mothers were diagnosed with an anxiety disorder at both time points. As expected, some mothers were diagnosed with more than one mental health disorder (*n* = 14). Seven mothers had a comorbid diagnosis of mood and anxiety or psychotic disorders at 3 or 6 months, six mothers had a comorbid diagnosis of mood and anxiety at both 3 and 6 months, while one mother had a comorbid diagnosis of mood, anxiety, and psychotic disorders at both time points.

**TABLE 2:** Maternal mental health diagnosis at 3‐ and 6‐month postnatal age

**Maternal mental health disorder diagnosis: 3 months**	**Maternal mental health disorder diagnosis: 6 months**	**Frequency of maternal mental health disorders at both time points (*n*)**
Mood disorder	Mood disorder	31
Psychotic disorder	Psychotic disorder	14
Anxiety disorder	Anxiety disorder	3
Mood disorder	Comorbid mood and anxiety disorder	1
Comorbid mood and anxiety disorder	Psychotic disorder	1
Comorbid mood and anxiety disorder	Mood disorder	4
Comorbid mood and psychotic disorder	Mood disorder	1
Comorbid mood and anxiety disorder	Comorbid mood and anxiety disorder	6
Comorbid mood and anxiety and psychotic disorder	Comorbid mood and anxiety and psychotic disorder	1

Mood disorders included major depressive disorder, bipolar disorder I and II, and borderline personality disorders. Anxiety disorders included generalized anxiety disorder, posttraumatic stress disorder, panic disorder, agoraphobia, and social phobia, while psychotic disorders included schizophrenia. A total of 23 mothers breastfed or used a combination of formula feeding and breastfeeding (mixed feeding) (Table 1). Eleven of these mothers were not using psychotropic medication at 6 months postnatal age, while 12 mothers used various classes of psychotropic medication while they were breastfeeding. Seven of these infants were exposed to various types and dosages of antipsychotic medication during breastfeeding, namely Haloperidol (*n* = 1), Olanzapine (*n* = 2), or Risperidone (*n* = 4). One infant was exposed to Promethazine. Four infants were exposed to selective serotonin reuptake inhibitors (SSRIs), namely Citalopram or Fluoxetine. One infant was exposed to a serotonin‐norepinephrine reuptake inhibitor (SNRI), namely Venlafaxine, and one infant was exposed to a tricyclic antidepressant (Amytriptyline).

### 3.3 Infant neurodevelopmental at 6 months post‐term

Table 3 presents the BSID‐III subtest composite and scaled scores, and the adjusted between‐group mean differences for infants with no exposure to maternal mental health disorders (comparison group; *n* = 35) and infants with exposure to maternal mental health disorders only at 3 and 6 months (two time points) (*n* = 62). Between‐group mean differences for the following three subgroups are also presented: infants with exposure to mood disorders at 3 and 6 months (two time points) (*n* = 31), infants with exposure to psychotic disorders at 3 and 6 months (two time points) (*n* = 14) and infants with exposure to comorbid disorders at 3 and 6 months (two time points) (*n* = 14). Since only three infants were exposed to an anxiety disorder at both the 3 and 6 months post‐term assessment, they were not included in the analysis.

**TABLE 3:** Adjusted mean differences in BSID‐III domain subtest composite and scaled scores at 6 months post‐term age according to maternal mental health disorders at 3 and 6 months

	**Comparison group *n* = 35** **^a^**	**Maternal mental health disorder exposure** ***n* = 62**			**Mood disorder exposure** **3 and 6 months** ***n* = 31**			**Psychotic disorder exposure** **3 and 6 months** ***n* = 14**			**Comorbid disorder exposure** **3 and 6 months** ***n* = 14**		
**BSID‐III subtest and scaled scores at 6 months**	**Mean (SE)**	**Mean (SE)**	**Mean difference (95% CI)**	*p*‐value*	**mean (SE)**	**Mean difference (95% CI)**	*p*‐ value**	**mean (SE)**	**Mean difference (95% CI)**	*p*‐ value***	**mean (SE)**	**Mean difference (95% CI)**	*p*‐ value****
**Cognitive composite score**	94.3 (1.2)	93.0 (.9)	−1.4 (−4.2–1.5)	.352	93.8 (1.2)	−.5 (−3.8–2.8)	.197	89.6 (1.8)	−**4.6** (−**8.7 to** −**.6)**	**.026**	95.0 (1.8)	.7 (−3.5–4.9)	.737
**Motor composite score**	109.8 (2.0)	107.7 (1.4)	−2.1 (−6.9–2.7)	.393	109.0 (2.0)	−.9 (−6.5–4.7)	.766	101.9 (3.0)	−**7.8** (−**14.6 to** −**.9)**	**.026**	113.0 (3.0)	3.3 (−3.7–10.2)	.364
**Fine motor scaled score**	12.4 (.4)	11.8 (.3)	−.6 (−1.5–.2)	.152	11.9 (.4)	−.6 (−1.6–.4)	.254	10.7 (.5)	−**1.8** (−**3.0 to** −**.5)**	**.005**	13.1 (.5)	.6 (−.6–1.9)	.323
**Gross motor scaled score**	11.0 (.4)	10.7 (.3)	−.4 (−1.4–.7)	.501	11.0 (.4)	−.01 (−1.2–1.2)	.99	9.9 (.7)	−1.1 (−2.6–.4)	.156	11.2 (.7)	.1 (−1.4 to −1.7)	.880
**Language composite score**	104.6 (1.2)	103.5 (.9)	−1.1 (−4.1–1.9)	.457	104.0 (1.3)	−.6 (−4.1–2.9)	.729	101.3 (1.9)	−3.3 (−7.6–1.1)	.139	105.2 (1.9)	.6 (−3.8–5.1)	.783
**Receptive scaled score**	9.2 (.2)	9.1 (.2)	−.1 (−.7–.5)	.684	9.2 (.3)	−.04 (−.7–.7)	.916	9.0 (.4)	−.2 (−1.1–.7)	.668	9.2 (.4)	.05 (−.8–1.0)	.907
**Expressive scaled score**	12.3 (.3)	12.1 (.2)	‐.2 (−.9–.5)	.555	12.1 (.3)	−.1 (−1.0–.7)	.732	11.4 (.4)	−.9 (−1.9–.1)	.087	12.5 (.4)	.3 (−.8–1.3)	.621
**Social‐emotional composite score**	111.0 (1.4)	113.0 (1.0)	2.1 (−1.3–5.5)	.235	113.2 (1.5)	2.2 (−1.8–6.2)	.276	111.1 (2.1)	.2 (−4.8–5.2)	.941	113.9 (2.2)	2.9 (−2.2–8.0)	.263
**Adaptive behavior composite score**	98.2 (1.5)	97.6 (1.1)	−.6 (−4.3–3.1)	.764	100.1 (1.5)	2.0 (−2.2–6.2)	.355	97.9 (2.3)	−.3 (−5.7–5.1)	.906	94.7 (2.3)	−3.5 (−9.0–2.0)	.209

All models were adjusted for gestational age.

Abbreviations: BSID‐III, Bayley Scales of Infant and Toddler Development®, Third Edition; CI, confidence interval; SE, standard error.

^a^ One missing BSID‐III subgroup measurement (social‐emotional composite score) for comparison group;.

Between‐group analysis (*p*‐value): ^*^ maternal mental health and comparison group; ^**^ mood disorders and comparison group; ^***^ psychotic disorders and comparison group; ^****^ comorbid group and comparison group.

Bold values indicate the significant.

Adjusted models revealed no significant mean differences on any of the BSID‐III domains between infants exposed to maternal mental disorders (*n* = 62) at 3 and 6 months and the comparison group. Correspondingly, no significant mean differences on any of the BSID‐III domains were found between infants with exposure to mood disorders (*n* = 31) and the comparison group, as well as between comorbid disorders (*n* = 14) and the comparison group (Table 3). None of the infants with exposure to mood disorders or co‐morbid disorders scored below average (<85) on any of the BSID‐III domains. Infants with exposure to psychotic disorders scored significantly lower in the cognitive domain (mean difference −4.6 [95% CI −8.7 to −.6]), the motor domain (mean difference −7.8 [95% CI −14.6 to −.9]), and the fine motor subscale (mean difference −1.8 [95% CI −3.0 to −.5]) than the comparison group (Table 3).

**FIGURE 1:**
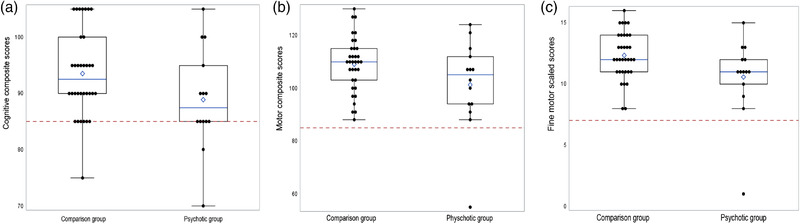
Box and whisker scatter plots for the cognitive composite scores (a), the motor composite scores (b), and the fine motor scaled scores (c) for the comparison and psychotic groups. The solid horizontal lines in the boxes represent the median values and diamonds represent the mean values. The vertical lines outside the boxes represent the upper quartiles (75th percentile) with maximum scores (upper horizontal lines) and lower quartiles (25th percentile) with minimum scores (lower horizontal lines). The dashed horizontal lines represent the cutoff for below‐average scores for the cognitive and motor domains (<85) (Figures 1a and 1b) and fine motor scaled scores (≤7) (Figure 1c). Individual scores for the infants are indicated with solid circles

The three box‐and‐whisker scatter plots in Figure 1 display the distribution of the cognitive composite scores (Figure 1A), the motor composite scores (Figure 1B), and the fine motor scaled scores (Figure 1C) of the psychotic group and the comparison group according to maternal mental health disorder exposure at 3 and 6 months. Two infants in the psychotic group and one infant in the comparison group scored below average (<85) on the cognitive composite scale (Figure 1a). The infant in the comparison group with a below‐average score (<85) on the cognitive composite scale was diagnosed with acute bacterial meningitis at 8 weeks post‐term. None of the infants in the comparison group scored below average on the motor composite and fine motor scales. One of the two infants in the psychotic group that scored below average (<85) on the cognitive composite scale also scored severely impaired (<70) on the motor composite scale (Figure 1b), as well as on the fine motor scale (≤7) (Figure 1c). The birth and post‐term medical history of both infants with a below‐average score (<85) on the cognitive composite scale were uneventful. However, one mother reported that she was smoking (tobacco) and consuming alcohol during pregnancy, while the other mother was using methamphetamine hydrochloride during pregnancy.

## 4 DISCUSSION

This study aimed to describe the early neurodevelopment of infants residing in low‐income urban and peri‐urban communities in Cape Town, South Africa, with exposure to persistent maternal mental health disorders. Adjusted models revealed that infants with exposure to persistent and concurrent maternal mental disorders (combined diagnoses; *n* = 62), as measured at 3 and 6 months postpartum, did not score significantly lower on the different domains and subscales of the BSID‐III at 6 months of age compared to infants with no exposure (comparison group). Maternal mental subgroup disorder analyses revealed no significant differences on any of the BSID‐III domains and subscales between infants with exposure to maternal mood disorders and infants with exposure to comorbid mood, anxiety, and psychotic disorders at 3 and 6 months compared to infants with no exposure. However, infants with exposure to psychotic disorders at 3 and 6 months scored significantly lower on the cognitive and motor domains and the fine motor subscale than infants with no exposure.

Previous studies conducted in HIC have reported that the persistence of maternal mental health symptoms throughout infancy has been associated with adverse outcomes in various developmental domains in young children (Gueron‐Sela et al., 2018; Ahun et al., 2017; Stein et al., 2014; Petterson & Albers, 2001; Brennan et al., 2000). This is especially true for mothers with inadequate social and partner support and mother–infant dyads living in lower socio‐economic status settings with exposure to higher levels of adversity (Gueron‐Sela et al., 2018; Stein et al., 2014; Petterson & Albers, 2001). Somewhat surprisingly, in the current study, infants exposed to persistent and concurrent mood disorders and to comorbid mood, anxiety, and psychotic disorders did not score significantly lower on any of the BSID‐III domains or subscales compared to infants with no exposure. Similar findings have been reported in studies conducted in LMICs regarding the impact of persistent maternal mental health disorders (measured at more than one time point). A large prospective study conducted in a socioeconomically disadvantaged rural district of Bangladesh found that maternal depressive symptoms diagnosed at 2–3 and 6–8 months postpartum were not associated with delayed motor development (Nasreen et al., 2013). A population‐based prospective study conducted in Ethiopia found that high levels of postnatal depression and anxiety, at more than one time point, were not significantly associated with infant cognitive, motor, and language outcomes on the BSID‐III at 12 months after adjusting for various confounders such as socio‐economic status, infant gender, or perceived social support (Servili et al., 2010). In Pakistan, a prospective study conducted in peri‐urban communities of Karachi found that a diagnosis of maternal depression and anxiety at three time points (1st‐, 2nd‐, and 6th‐month post‐term) had no significant impact on fine and gross motor development of infants at 6 months. They did, however, find that infants exposed to persistent depression and anxiety showed delayed cognitive and emotional development at 6 months, especially if they were stunted (Ali et al., 2013). Furthermore, exposure to persistent postpartum anxiety and depression and stunted growth were significantly associated with delayed development at 12 and 18 months in all five developmental domains (Ali et al., 2013). In LMICs, children of mothers with persistent depression (multiple episodes) are particularly at risk of stunting or being underweight (Stein et al., 2014), which in turn are important risk factors for impaired neurodevelopment (Abubakar et al., 2010). The fact that none of the infants in our study were underweight (WAZ of −2 or below) may have served as a protective factor, rendering them less vulnerable to persistent maternal mental health disorders.

Another potential explanation for why we did not find an association between persistent exposure to maternal mood and comorbid disorders and adverse infant neurodevelopment is because we focused on the early impact of persistent and concurrent effects of maternal mental health disorders on infant neurodevelopment. The first 6 months post‐term is the time when maternal mental disorders, specifically postpartum depressive symptoms, are most prevalent (Stuart‐Parrigon & Stuart, 2014). Evidence from high‐income and LMICs suggests that prolonged exposure to maternal depression and anxiety throughout the first 12 months and beyond have been associated with adverse neurodevelopmental outcomes, particularly lower cognitive performance, in toddlers and older children (Netsi et al., 2018; Ali et al., 2013; Cornish et al., 2005). Therefore, studies assessing the impact of maternal mental health during early infancy (i.e., first 6 months post‐term) are less likely to find an association. Since the present study is nested within a larger ongoing longitudinal cohort design, repeated measures will be used to assess maternal mental health and the neurodevelopmental outcome of the infants until preschool age.

Although the infants that were exposed to maternal psychotic disorders scored significantly lower on the cognitive and motor domains and fine motor subscale of the BSID‐III, the majority presented with mean values within the average performance range (composite score: 85–115; scaled score: 8–12) and, therefore, did not demonstrate a clinically significant developmental delay. Only two infants exposed to maternal psychotic disorders scored below average on the cognitive domain and one of these infants also scored severely impaired on the motor domain and fine motor subscale. Although the birth and medical history of these infants were uneventful, they were exposed to smoking and alcohol, and methamphetamine hydrochloride prenatally. To our knowledge, there are no published reports on maternal psychosis in the context of infant neurodevelopment in Africa, and research on this topic in the rest of the world is extremely limited. In fact, a recent scoping review of the literature on schizophrenia and motherhood found that data on neurodevelopmental outcomes of offspring of mothers with schizophrenia are too scarce to draw any conclusion (Gentile & Fusco, 2019). Earlier observations in low‐income, single‐parent families in the United States of America found that infants exposed to maternal schizophrenia scored lower on the Mental Development Index of the BSID‐I (mean composite score of 85) than infants of depressed mothers (mean composite score of 99) and infants of well mothers (mean composite score of 97). However, these cognitive delays, reported in infancy, were transient and disappeared in later childhood (Goodman, 1987). Likewise, short‐term and transient delays in the motor, cognitive, social‐emotional, and adaptive behavioral domains of the BSID‐III at 6 months follow‐up were reported in infants born to mothers with schizophrenia and treated with an atypical antipsychotic throughout pregnancy in China (Peng et al., 2013).

The lower cognitive and fine motor scores of infants exposed to maternal psychotic disorders in the current study may be explained by the psychosocial environments that mothers with postnatal psychosis tend to provide. Maternal postnatal psychotic disorders are associated with maladaptive and detached maternal behavior, which may have a negative effect on infant development (Wan et al., 2008). Compared to mothers with other psychiatric diagnoses, schizophrenic mothers have been described as more remote and self‐absorbed, verbally and behaviorally intrusive, insensitive and unresponsive, and displaying less warmth, while their 4‐month‐old infants were less attentive and demonstrating avoidance during maternal–infant interactions (Wan et al., 2008; Wan et al., 2007; Riordan et al., 1999). Furthermore, severe mental illness such as schizophrenia is associated with more deprivation through social adversity and insufficient partner and social support (Glangeaud‐Freudenthal et al., 2014). Therefore, infants and children born to mothers with schizophrenia have fewer resources to draw on to develop resilience. Another factor that may explain the lower cognitive and fine motor scores is that seven of the 14 infants with maternal psychotic disorder exposure were also exposed to various types and dosages of antipsychotic medication during breastfeeding. Six of the seven infants were exposed to second‐generation (atypical) antipsychotics. A systematic review found that second‐generation antipsychotics during the lactation period are related to minimal and transient effects in a very small portion of exposed infants (Uguz, 2016). It is, however, difficult to disassociate the contribution of antipsychotic medication and postnatal maternal psychosis on the neurodevelopmental outcome of exposed infants in the current study.

Our study's strengths include the use of structured diagnostic psychiatric interviews, with a clinical diagnosis according to defined DSM‐V criteria (Regier et al., 2013), conducted by a senior psychiatrist. The neurodevelopmental assessments were conducted by a pediatric physiotherapist blinded to maternal history and infant exposure. We used a longitudinal design to assess maternal mental health at two time points and can, therefore, infer a causal link between maternal mental health status and infant neurodevelopment. Although we have included an ethnically, linguistically, and culturally heterogeneous sample, our findings have limited generalizability due to the inclusion of persistent maternal psychopathology with findings from a single medical center. To the best of our knowledge, this study was the first to provide insights into the early neurodevelopment of infants exposed to maternal psychotic disorders in Africa. It should be borne in mind that findings are based on small sample sizes of the different psychiatric diagnostic subgroups; therefore, our findings should be regarded as preliminary. However, the small sample of specifically the psychotic disorder group reflects the relative rarity of cases of this kind. A total of 12 mothers used various classes of psychotropic medication while they were breastfeeding. In keeping with best practice recommendations regarding teratogens in medication during breastfeeding, all mothers were on the lowest possible dose of required medications at the time of the study. Due to the small number of mothers using psychotropic medication while they were breastfeeding, it was not possible to analyze the contribution of psychotropic medication on the neurodevelopment of exposed infants.

The fact that infants exposed to persistent maternal mood and comorbid disorders at 3 and/or 6 months did not score significantly lower on the BSID‐III domains may be due to protective factors in the infants’ environment, which may have operated as potential moderators. Prior studies identified protective factors that had a positive influence on early child development in the presence of poor maternal health (McDonald et al., 2016; Huang et al., 2014; Di Cesare et al., 2013). These factors included dependable maternal social support structures, stable and healthy interpersonal relationships, higher maternal optimism and education, less difficulty balancing work and family responsibilities, and establishing good sleep habits for the infant (McDonald et al., 2016; Huang et al., 2014; Di Cesare et al., 2013). For example, in the current study, a partner or family member sharing the same household may have moderated the potential adverse effects of the maternal mental disorder by offering support for the mother and provided a sensitive and responsive caregiving environment for the infant. Likewise, the infants (13.3% in the exposure group and 11.8% in the comparison group) that were attending a crèche or were cared for by their grandmothers or nannies during the day, could have been exposed to a more nurturing environment. We were however not able to measure social support, or lack thereof, that the mother–infant dyads received. We also lack observations on maternal behaviors, mother–infant interactions, and maternal infant‐handling practices, making it difficult to understand and explain the pathways through which maternal mental health status is associated with infant neurodevelopment. Another limitation of our study was that we did not control for prenatal mental health and/or distinguish the impact of persistent mental health disorders prior to the postpartum period. The present study was not designed to analyze possible mechanisms underlying the effect of maternal mental health disorders on infant neurodevelopment. Therefore, future research should explore if the infant's home environment, maternal behavior, mother–infant interactions, and social support structures may moderate the effect of persistent or brief episodes of maternal mental health disorders on the infant's short‐ and long‐term neurodevelopment.

## 5 CONCLUSION

In summary, our study cohort represents mothers and their infants who face substantial mental health and socio‐economic challenges. While maternal mental health disorders and the adverse consequences for child health and development have received substantial research attention in HICs, relatively little work has been done in Africa. The current study is among the first to report on the early neurodevelopmental outcome among infants exposed to a clinical diagnosis of maternal mental health disorders. This study has shown that despite being exposed to substantial maternal mental health adversities and living in poor socio‐economic status settings, the majority of infants in our study did not score significantly lower on the different domains and subscales of the BSID‐III at 6 months compared to infants with no exposure. However, infants with exposure to psychotic disorders at 3 and 6 months scored significantly lower on the cognitive and motor domains and the fine motor subscale compared to infants with no exposure and the longer‐term implications need to be assessed. These novel data provide an important contribution to the scientific literature given the lack of published data especially in the field of maternal psychotic disorders in Africa. Since maternal–infant psychosocial risk factor profiles in Africa differ from those in HICs, future research should focus on longitudinal assessment of mothers with mental health disorders, including psychotic disorders, and the neurodevelopmental outcome of their children beyond the first postnatal year. Future research should address a critical gap by exploring how the infant's home environment, maternal behavior, mother‐infant interactions, and social support structures may impact the infant's short‐ and long‐term neurodevelopment. Understanding which risk factors lead to poorer neurodevelopment and which protective factors build resilience in infants exposed to maternal mental health disorders is essential for intervention policy design to optimize early child development in Africa.
